# Clinical Features and Novel Pathogenic Variants of Chinese Patients With McLeod Syndrome and Chorea‐Acanthocytosis

**DOI:** 10.1002/mgg3.70015

**Published:** 2024-09-26

**Authors:** Hao Yu, Ling Li, Xiaoyan Li, Haipeng Liu

**Affiliations:** ^1^ Department of Medical Genetics and Center for Rare Diseases, Second Affiliated Hospital Zhejiang University School of Medicine and Zhejiang Key Laboratory of Rare Diseases for Precision Medicine and Clinical Translation Hangzhou Zhejiang China; ^2^ Department of Neurology, Zhoushan Hospital Wenzhou Medical University Zhoushan Zhejiang China; ^3^ Research Centre for Intelligent Healthcare, Faculty of Health and Life Sciences Coventry University Coventry UK

**Keywords:** chorea‐acanthocytosis, McLeod syndrome, *VPS13A*, *XK*

## Abstract

**Background:**

McLeod syndrome (MLS) and chorea‐acanthocytosis (ChAc) are exceedingly rare diseases characterized by a variety of movement disorders including chorea, dystonia, and Parkinsonism. Genetic analysis plays a key role in early and accurate diagnosis, but relevant variants are still under investigation. This study aims to explore new pathogenic variants in Chinese patients with MLS and ChAc and to conduct a comprehensive analysis of the clinical heterogeneity among these patients.

**Methods:**

Eighteen Chinese patients who presented with choreatic movements with negative *HTT* genetic testing were identified and underwent targeted next‐generation sequencing, verified by Sanger sequencing.

**Results:**

Two novel *XK* variants (c.970A>T, c.422_423del) were identified in three index MLS patients and six novel *VPS13A* variants (c.9219C>A, c.3467T>A, c.4208dup, c.9243_9246del, c.5364del, c.556‐290_697‐483del) in five index ChAc patients. One copy number variant of *VPS13A* (g.79827595_79828762del/c.556‐290_697‐483del) was firstly described in Chinese population.

**Conclusion:**

As the currently largest descriptive study of MLS and ChAc patients in China, this study expands on the clinical and genetic spectrum of *XK* and *VPS13A*, contributing to the clinical diagnosis of MLS and ChAc.

## Graphical abstract

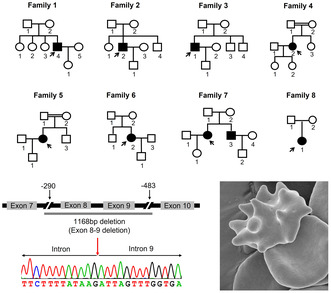

## 1 Introduction

McLeod syndrome (MLS) and chorea‐acanthocytosis (ChAc) are two rare neurodegenerative genetic diseases (Kaestner 2023; Walker and Danek 2021). MLS shares a variety of features with ChAc, such as presence of acanthocytes in blood and degeneration of the basal ganglia (Peikert and Danek 2023), specifically the caudate nucleus and putamen (Roulis et al. 2018). The characteristic phenotypes of MLS and ChAc can resemble Huntington's disease (HD) and can involve psychiatric and cognitive symptoms, and behavioral changes, as well as a variety of movement disorders, for example, chorea, dystonia, and parkinsonism (Jung et al. 2004; Kaestner 2023; Peikert et al. 2002).

For both MSL and ChAc, neuroimaging often shows atrophy of the basal ganglia and dilated anterior horns of the lateral ventricles (Jung et al. 2004; Peikert et al. 2002; Peikert, Hermann, and Danek 2022). In laboratory tests, elevated creatine kinase (CK) level and increased acanthocyte count were observed on peripheral blood smears (Jung et al. 2004; Peikert et al. 2002). Some biomarkers in blood tests may help in the diagnosis, including serum neurofilament protein light chain, acanthocyte sedimentation rate, and erythrocyte sedimentation rate (Darras et al. 2021; Peikert et al. 2020; Rabe et al. 2021). The diagnosis of MLS relies on specific immunohematologic tests, where McLeod phenotype is characterized by the absence of the Kx antigen and weakened or absent Kell antigens (Jung et al. 2004). Individuals with the McLeod phenotype have rare blood groups due to the absence of the Kx antigen, which increases transfusion risks, making autologous banked donations preferable when feasible (Jung et al. 2004; Roulis et al. 2018).

Despite the diagnostic value of clinical manifestations and biomarkers, accurate diagnosis of MLS and ChAc depends on genetic analysis. However, the rarity of these conditions does not allow for conclusive genotype–phenotype correlation, which makes fine‐grained classification challenging. MLS and ChAc share a wide range of clinical features, and recent studies have focused on identifying the genetic mutations that cause them (Roulis et al. 2018), which will help to broaden the genetic spectrum and elucidate the underlying mechanisms of the disease. MLS is inherited in an X‐linked way with pathogenic variants of *XK* gene, while ChAc is an autosomal recessive disorder caused by pathogenic variants in *VPS13A*. It is estimated that there are several hundred MLS patients and approximately 1000 ChAc patients worldwide (Dulski et al. 2016). As the most populous country, China holds significant potential for discovering new variants of these syndromes.

To address this gap, we collected 18 unrelated Chinese patients and performed targeted next‐generation sequencing (NGS) for the diagnosis of MLS or ChAc. Two novel *XK* variants and six novel *VPS13A* variants were identified.

## 2 Methods

### 2.1 Standard Protocol Approvals, Registrations, and Patient Consents

This study was approved by the Ethics Committee of the Second Affiliated Hospital of Zhejiang University School of Medicine. Written informed consent was obtained from all patients participating in the study.

### 2.2 Subjects

Eighteen unrelated patients who presented with choreatic movements and their family members were recruited between June 2015 and December 2023. Inclusion criteria: (1) Neurological examination and clinical evaluation by at least two senior neurologists to confirm the presence of choreatic movements. (2) Negative results in the *HTT* gene test to rule out HD. Exclusion criteria: (1) comorbidity with delayed movement disorders, hepatomegaly, Parkinson's disease, motor neuron disease; (2) accompanied by other etiologies that may cause chorea, such as infectious and post‐infectious chorea, autoimmune chorea, post vascular disease chorea, multisystemic and metabolic chorea, and so forth; (3) comorbidity with severe cardiac, renal, hepatic, hematological, autoimmune disorders, malignant neoplasm.

### 2.3 General Information

Clinical data of the patients were collected: age of onset, clinical phenotype, and family history of each patient were collected. Blood test results: creatine kinase (CK), CK‐myocardial band (CK‐MB), lactate dehydrogenase (LDH), alanine aminotransferase (ALT), glutamine aminotransferase (AST), and hemoglobin (Hgb). Electrophysiological examination: nerve conduction velocity/electromyography (NCV/EMG) after patient's consent. Radiological examination: cranial magnetic resonance imaging (MRI) performed after admission. Imaging data were obtained and first analyzed by a radiologist with extensive experience in MRI image interpretation, and another neuroradiologist with more than 10 years of experience in interpreting physician brain MRI images subsequently reviewed the results and finalized the report. The radiologists were blinded to the participant group. In patients with a high clinical suspicion of MLS, blood samples were sent to the Zhejiang Blood Centre China for antigen analysis of the Kell blood group system antigen.

### 2.4 Acanthocyte Examination

Blood was collected into the tube containing ethylene‐diamine‐tetracetic acid (EDTA) and examined by scanning electron microscopy (SEM). Red cells were fixed in a 1% solution of glutaraldehyde and stored at 4°C until the time of examination. The red cells were absorbed onto tissue paper and gold‐coated with an Emscope Sputter Coater. The morphology of red cells was then examined in a Nova nano 450 electron microscope operating at 5 kV. Erythrocyte morphology was assessed by counting ≥ 800 cells (50 erythrocytes for each field of view at a magnification of 3000×), where all counts were triplicate with the mean used for the analysis of acanthocyte rate (Ciccoli et al. 2013). Normal red cells had a biconcave disk shape. Contracted red cells with a number of irregularly spaced thorny surface projections were defined as acanthocytes (Yu et al. 2022).

### 2.5 Genetic Analysis

Genomic DNA was extracted from peripheral EDTA‐treated blood using Blood Genomic Extraction Kit (Qiagen, Hilden, Germany). The NGS used in the study included whole‐exome sequencing (WES) or a customized panel which covered 54 genes for chorea and dystonia, including *VPS13A*, *XK*, *PRNP*, 10 genes of neurodegeneration with brain iron accumulation (NBIA), four genes of primary familial brain calcification (PFBC), and other 37 genes of hereditary dystonia (Table S1). A detailed protocol of NGS has been reported in our previous study (Liu et al. 2017). Sanger sequencing was performed on an ABI 3500xl Dx Genetic Analyzer (Applied Biosystems, Foster City, USA) to verify the filtered potential variants (Dong et al. 2016). Co‐segregation analysis was carried out in families with genetic variants. The locations of sequence variation were reported using the National Center for Biotechnology Information's (NCBI) Reference Sequence (RefSeq) database. Specifically, the accession numbers NM_021083.3 and NM_033305.3 (NC_000009.11) correspond to the respective latest versions of the *XK* and *VPS13A* gene sequences in the RefSeq database. Sorting intolerant from tolerant (SIFT) and Polymorphism Phenotyping v2 (PolyPhen‐2) were used to predict the pathogenicity of the identified variants. Patients with a genetically confirmed diagnosis of NA were followed up regularly until November 2023 by telephone.

## 3 Results

### 3.1 Identification of Novel XK and VPS13A Variants

**FIGURE 1:**
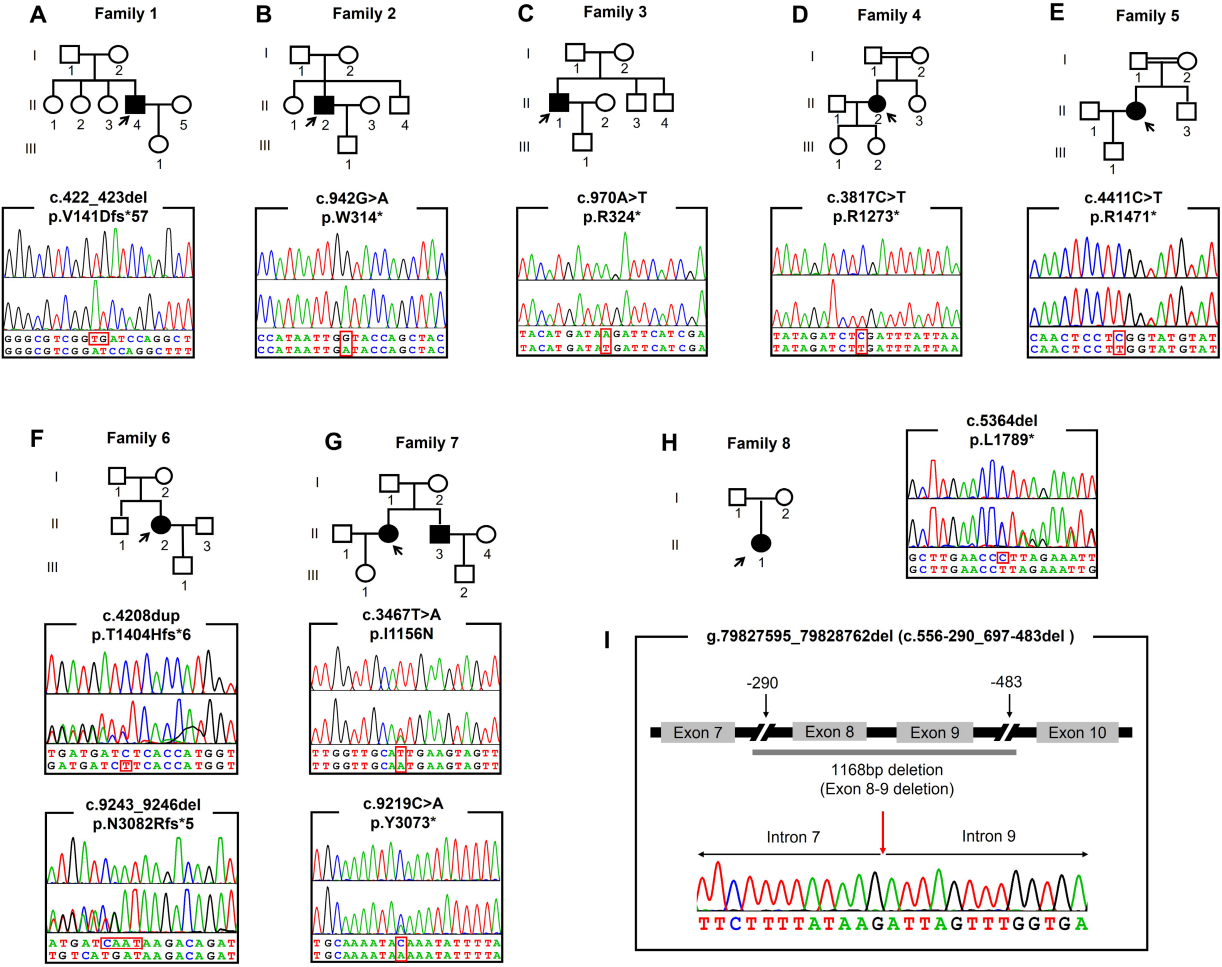
(A–H) Pedigree chart of index families and chromatogram of novel variants detected in each family. Squares indicate males; circles indicate females; the black symbols indicate affected individuals; arrows indicate the probands. The upper panel in chromatogram depicts the reference sequence. The lower panel represents heterozygous mutated sequence. (I) The breakpoint analysis of the copy number variants in Family 8. The upper panel depicts the schematic diagram of the exons 8–9 deletion on DNA level. The lower panel represents the mutated sequence verified by Sanger sequencing.

After performing NGS and Sanger sequencing, genetic variants were identified in eight of the 18 individuals presenting with choreatic movements. Therefore, this study included three patients with MLS and five with ChAc. Additionally, genetic testing was conducted on relatives of some patients presenting with choreatic movements who agreed to participate. Among the MLS patients, three hemizygous variants within the *XK* gene were found in three index patients (Figure 1A–C). All three variants are either nonsense or frameshift, and two are novel variants (c.970A>T, c.422_423del). They are all absent in gnomAD and our NGS database containing 1000 Chinese matched controls. According to American College of Medical Genetics and Genomics (ACMG) standards (Richards et al. 2015), they are classified as either pathogenic or likely pathogenic (Table 1).

**TABLE 1:** The identified variants of patients with McLeod syndrome and chorea‐acanthocytosis.

Gene	Case no.	RefSeq version	Nucleotide (Amino acid) change	Exon	Variant type	gnomAD frequency	ACMG/AMP classification (criteria)
*XK*	1	NM_021083.3	c.422_423del (p.V141Dfs*57)	2	Hemi	0	P (PVS1, PM2_Supporting, PP4)
*XK*	2	NM_021083.3	c.942G>A (p.W314*)	3	Hemi	0	P (PVS1_Strong, PS1, PM2_Supporting)
*XK*	3	NM_021083.3	c.970A>T (p.R324*)	3	Hemi	0	LP (PVS1_Strong, PM2_Supporting, PP4)
*VPS13A*	4	NM_033305.3	c.3817C>T (p.R1273*)	34	Hom	8e‐06	P (PVS1, PM2_Supporting, PP4)
*VPS13A*	5	NM_033305.3	c.4411C>T (p.R1471*)	37	Hom	4e‐06	P (PVS1, PM2_Supporting, PP4)
*VPS13A*	6	NM_033305.3	c.4208dup (p.T1404Hfs*6)	36	Het	0	LP (PVS1, PM2_Supporting)
*VPS13A*	6	NM_033305.3	c.9243_9246del (p.N3082Rfs*5)	69	Het	0	LP (PVS1, PM2_Supporting)
*VPS13A*	7	NM_033305.3	c.3467T>A (p.I1156N)	32	Het	4e‐06	LP (PM2_Supporting, PM3, PP1, PP3, PP4)
*VPS13A*	7	NM_033305.3	c.9219C>A (p.Y3073*)	69	Het	0	P (PVS1, PM2_Supporting, PP1, PP4)
*VPS13A*	8	NM_033305.3	c.5364del (p.L1789*)	42	Het	0	P (PVS1, PM2_Supporting, PP4)
*VPS13A*	8	NC_000009.11 /NM_033305.3	g.79827595_79828762del /c.556‐290_697‐483del	8–9	Het	0	LP (PVS1_Moderate, PM2_Supporting, PM3_Strong, PP4)

*Note:* The general recommendations for using the ACMG/AMP criteria can be found in Clinical Genome Resource website (www.clinicalgenome.org/working‐groups/sequence‐variant‐interpretation).

Abbreviations: ACMG/AMP, American College of Medical Genetics and Genomics and Association for Molecular Pathology; gnomAD, Genome Aggregation Database; Hemi, hemizygous; Het, heterozygous; Hom, homozygous; LP, likely pathogenic; P, pathogenic; RefSeq, NCBI Reference Sequence database.

Eight variants within *VPS13A* gene were detected in five index patients of ChAc, three of whom carried compound heterozygous variants (c.9219C>A, c.3467T>A, c.4208dup, c.9243_9246del, c.5364del, g.79827595_79828762del). In these three families, the father of Case 6 carries the heterozygous c.9243_9246del variant, the mother carries the heterozygous c.4208dup variant, the daughter of Case 7 carries the heterozygous c.3467T>A variant, and the father of Case 8 carries the heterozygous c.5364del variant. Two homozygous variants were detected in other two families (c.3817C>T, c.4411C>T) (Figure 1D–I). Among these variants, six are either nonsense or frameshift, one is missense, and one is copy number variant. Six variants are novel (c.9219C>A, c.3467T>A, c.4208dup, c.9243_9246del, c.5364del, c.556‐290_697‐483del). The copy number variant was firstly described in Chinese population. All variants are at extremely low frequency in gnomAD and absent in our NGS database. The missense variant c.3467T>A was predicted to be deleterious by SIFT and PolyPhen‐2. According to ACMG standards, all variants are classified as either pathogenic or likely pathogenic (Table 1).

### 3.2 Clinical Features of Eight Genetically Diagnosed Patients

**FIGURE 2:**
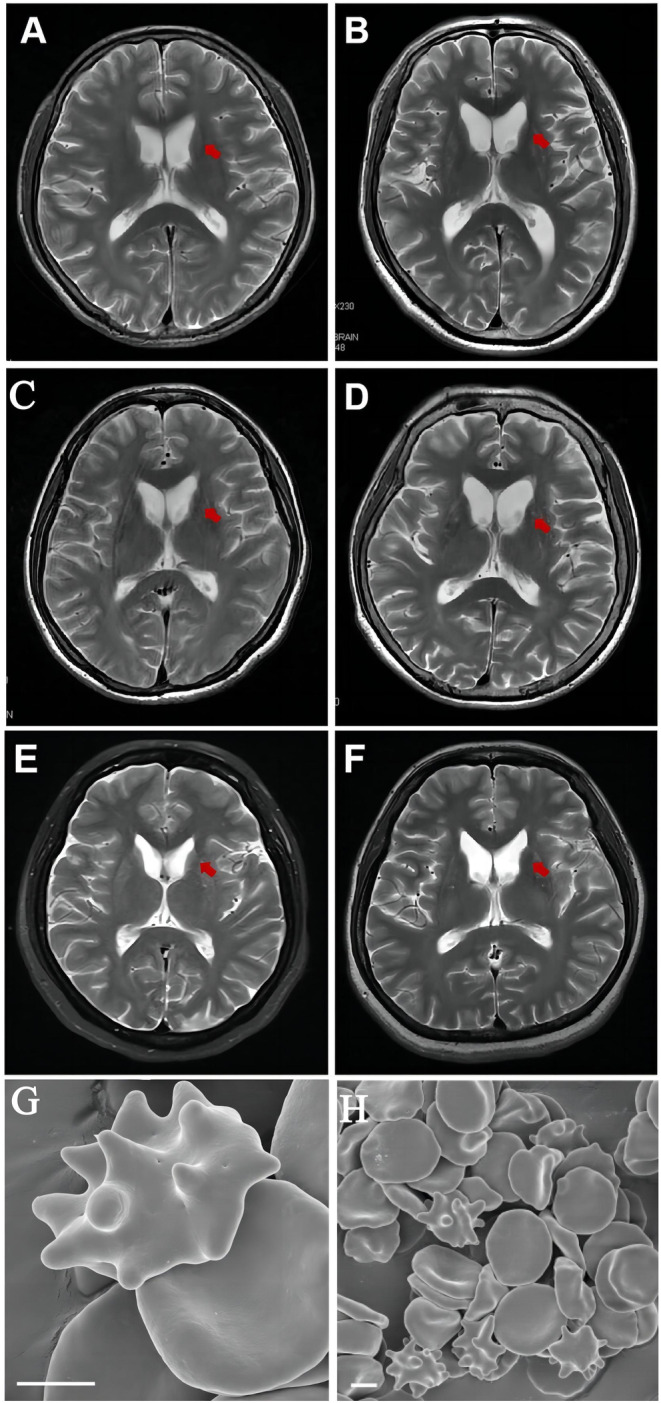
Clinical features of six genetically diagnosed patients. (A–F) Brain MRI of Case 1 (A), Case 3 (B), Case 5 (C), Case 6 (D), Case 7 (E) and Case 8 (F). Note the atrophy of caudate and the enlarged anterior horns of lateral ventricles. (G,H) Pictures of acanthocytes in peripheral blood of Case 7 by scanning electron microscopy. Scale bar: 2 μm.

The detailed clinical features of three MLS and five ChAc patients are summarized in Table 2. Three MLS index patients were all males with no family history of similar symptoms. Five ChAc index patients were all females, of whom two were from consanguineous marriage and two had a positive family history. Eight patients had mild to moderate elevated CK. One MLS case (Case 2) showed gait disturbance, mild involuntary movements and markedly elevated CK, which made it confused with myopathy at first whilst the diagnosis was confirmed after a second admission. All patients had diminished or absent tendon reflexes, and four of them agreed to undergo NCV/EMG and the results showed severe axonal neuropathy in two MLS patients, and normal NCV with mild chronic neurogenic EMG changes in two ChAc patient. MRI of the brain in six patients showed atrophy of either caudate or putamina (Figure 2A–F). Except for one case undetected, the acanthocyte rate of peripheral blood in seven patients was between 16%–32%, which was easily recognized in electron microscopy (Figure 2G,H). Case 2 underwent Kell blood group phenotyping at the blood center after the second admission. The results indicated the absence of K, Kpa, and Kpb antigens, along with a weak positive reaction for the k antigen on his red blood cells, which is consistent with the McLeod phenotype. Regrettably, the assessment of Kx antigen manifestation could not be executed owing to the non‐availability of antibodies.

**TABLE 2:** Clinical features of three McLeod syndrome and five chorea‐acanthocytosis patients.

Variable	Case 1	Case 2	Case 3	Case 4	Case 5	Case 6	Case 7	Case 8
Diagnosis	MLS	MLS	MLS	ChAc	ChAc	ChAc	ChAc	ChAc
Gender/Age	Male/47	Male/41	Male/57	Female/35	Female/43	Female/41	Female/61	Female/31
Age of onset	26	37	53	Early of 20s	33	39	50	27
Family history	—	—	—	—	+	—	+	—
Primary symptoms	Involuntary tongue and limb movements	Gait disturbance	Involuntary limb movements	Involuntary tongue and limb movements	Gait disturbance	Dysarthria	Involuntary tongue and limb movements	Involuntary limb movements
Chorea movements	Generalized	Mild	Generalized	Generalized	Generalized	Mild	Generalized	Generalized
Dysarthria	+	—	+	+	+	+	+	+
Orofacial dyskinesias	+	—	+	+	+	+	+	+
Tongue and lip biting	—	—	—	+	+	—	—	+
Seizure	Generalized	—	—	Generalized	—	—	—	—
CK (< 164 U/L)	753	4469	345	332	394	821	252	1616
CK‐MB (< 24 U/L)	39	99	35	22	39	26	20	29
LDH (120–250 U/L)	230	419	355	159	230	190	192	267
ALT (< 45 U/L)	24	90	119	14	27	16	28	22
AST (< 35 U/L)	31	85	29	18	26	24	28	44
Hgb (113–151 g/L)	145	157	132	121	145	111	121	143
NCV/EMG	Severe axonal neuropathy	Severe axonal neuropathy	NA	NA	Mild chronic neurogenic changes	Mild chronic neurogenic changes	NA	NA
Acanthocyte ratio (%)	18%	22%	NA	32%	16%	28%	30%	20%
MRI	Atrophy of the heads of the caudate nuclei	NA	Atrophy of putamina and caudate	Atrophy of putamina and caudate	Atrophy of putamina and caudate	Atrophy of putamina and caudate	Atrophy of putamina and caudate	Atrophy of putamina and caudate
Outcome	Rapid progress	Slow progress	Death	Rapid progress	Rapid progress	Slow progress	Death	Rapid progress

Abbreviations: −, negative; +, positive; ALT, alanine aminotransferase; AST, aspartate aminotransferase; ChAc, chore‐acanthocytosis; CK, creatine kinase; CK‐MB, creatine kinase‐MB (CK‐MB); Hgb, hemoglobin; LDH, lactate dehydrogenase; MLS, McLeod syndrome; NA, not available; NCV/EMG, nerve conduction velocity/ electromyography.

### 3.3 Outcome

Most cases had a poor prognosis. Cases 1, 4, 5, 7, and 8 progressed progressively to a bedridden state, unable to live independently, within 3 years of diagnosis. Two patients died, Case 3 from a fall in a rice paddy 1 year after diagnosis and Case 8 from pneumonia. Cases 2 and 6 had relatively slow progression and mild symptoms and could still live independently.

## 4 Discussion

MLS and ChAc are exceedingly rare and are often mistaken for each other in clinical practice (Peikert and Danek 2023) due to similarities in clinical phenotypes, laboratory tests, and neuroimaging. A thorough clinical evaluation, including history, physical examination, laboratory and imaging tests, and genetic testing, is essential for the accurate classification of these disorders and the exclusion of other conditions. The diagnosis of MLS and ChAc primarily relies on identifying mutations in the *XK* and *VPS13A* genes. The current HumanGene Mutation Database (HGMD, v2023.4) includes 53 mutations in the *XK* gene and 180 in the *VPS13A* gene. These mutations mainly consist of missense/nonsense variants, splicing variants, small deletions/insertions/indels, and gross deletion/insertion/complex rearrangements. Such mutations often result in premature protein translation termination or alternative splicing during transcription, leading to truncated or absent proteins that impair protein function (Ying et al. 2023).

We identified a surprisingly high number of *XK* and *VPS13A* variants in 18 *HTT*‐negative patients with choreatic movements, including three variants in the *XK* gene and eight in the *VPS13A* gene. The proportion of MLS and ChAc patients found in our study (8/18) was significantly higher compared to 0/28 in France (Mariani et al. 2016) and 3/19 in Mexico (Ramírez‐García et al. 2022), where HD phenotype patients were *HTT*‐negative. This discrepancy may be mostly due to potential patient selection bias. Our department is a regional center of rare diseases in Zhejiang Province. Therefore, many patients are referred from other hospitals. The participation of interdisciplinary teams in first‐line diagnosis might also contribute to the enrichment of characteristic rare diseases in our department. Meanwhile, ethnicity may also contribute to this difference. In accordance with previously reported variants (Chen et al. 2023; Huang et al. 2022; Man, Yuen, and Fu 2014; Shen et al. 2017; Xia et al. 2022; Yi et al. 2018), we found that no variant has been found in more than one family, thus there is no hotspot variant, which indicates a high level of genetic variability in the Chinese population. We discovered two novel *XK* variants in three index MLS patients and six novel *VPS13A* variants in five index ChAc patients. In addition, a copy number variation in *VPS13A* (g.79827595_79828762del/c.556‐290_697‐483del) was identified for the first time in the Chinese population (Spieler et al. 2020). As the currently largest descriptive study of MLS and ChAc patients in China, our research expands on the clinical and genetic spectrum of *XK* and *VPS13A*, contributing to the clinical diagnosis of MLS and ChAc.

### 4.1 Molecular Mechanisms of MLS and ChAc

The clinical similarity between MLS and ChAc diseases suggests that they share some common pathophysiology (Peikert, Hermann, and Danek 2022). Chorein is present in mature erythrocytes and is a product of the *VPS13A* gene, which is partially or completely absent in the erythrocytes of patients with ChAc (Huang et al. 2022). There is a molecular interaction between *XK*, the McLeod protein, and chorein, the product of the *VPS13A* gene, in which *XK* forms a complex with chorein/VPS13A, functioning as a bulk lipid transporter at various membrane contact sites (Peikert, Hermann, and Danek 2022). *XK‐VPS13A*‐mediated perturbation of phospholipids at the plasma membrane plays a crucial role in maintaining homeostasis in the nervous and erythrocyte systems (Ryoden and Nagata 2022). Dysregulation of the *VPS13A‐XK* complex, with impaired bulk lipid transport, is a common pathophysiological basis for both MLS and ChAc disorders (Miltenberger‐Miltenyi et al. 2023; Park and Neiman 2020; Peikert, Hermann, and Danek 2022). Conditions associated with *VPS13A* and *XK* genes may be pivotal to a novel disease paradigm, that is, bulk lipid transfer disorder (Peikert and Danek 2023).

### 4.2 Different Clinical Phenotypes of MLS and ChAc

The phenotypic similarities between MLS and ChAc diseases can lead to confusion, but some clues may help to differentiate them. MLS is inherited in an X‐linked way and ChAc in an autosomal recessive manner. Therefore, the gender and family history can give important clues. Larger deletions in the *XK* gene may also involve chronic granulomatous disease, dystrophinopathy, retinitis pigmentosa and ornithine transcarbamylase deficiency (Peikert, Hermann, and Danek 2022). In clinical manifestations, orofacial dystonia is more frequently presented in ChAc, particularly self‐mutilating tongue and lip biting, which is extremely rare in MLS (Peikert et al. 2002; Peikert, Hermann, and Danek 2022; Shen et al. 2017). MLS has a longer course, with a higher prevalence of arrhythmias and dilated cardiomyopathy, and more severe cardiac involvement (Jung et al. 2004; Peikert, Hermann, and Danek 2022; Quick et al. 2021). ChAc may show considerable phenotypic variability, even within the same family, regarding age, presenting symptoms, development of additional symptoms, and course of the disease, and 10% of patients are suicidal (Roulis et al. 2018; Walker et al. 2019). The underlying reasons for this diversity in clinical manifestations remain to be fully explored.

### 4.3 Laboratory and Neurophysiological Examinations

Most patients have elevated CK and abnormal liver function, and CK levels appear to be higher in patients with MLS than in ChAc (Jung et al. 2004; Peikert et al. 2002; Wu et al. 2017). The acanthocyte account in peripheral blood may range considerably within 5%–50% in ChAc and 8%–30% in MLS, making it unreliable as an index for differentiation (Jung et al. 2004; Peikert et al. 2002). Sensory and motor neuropathy are typical manifestations of ChAc and MLS, and nerve conduction velocity findings are usually indicative of axonal loss (Ghodsinezhad et al. 2024; Peikert, Hermann, and Danek 2022). Previous studies have shown that the majority of muscle biopsies in patients with ChAc show a neurogenic phenotype, with minor muscle lesions observed in a few cases (Vaisfeld et al. 2021). In comparison, MLS is more often considered a histological manifestation of less severe neurogenic lesions and primary myopathies (Vaisfeld et al. 2021). Moreover, MLS patients can have muscle complaints with mild involuntary movements, which often lead to a confusion with myopathy (Chen et al. 2014; Man, Yuen, and Fu 2014). A study has shown that the lack of *XK* expression in MLS correlates with type 2 fiber atrophy and suggests that the *XK* protein is crucial for maintaining normal muscle structure and function, potentially explaining the myopathy observed in MLS patients (Jung, Russo, et al. 2001).

### 4.4 Neuroimaging Features of MLS and ChAc

Neuroimaging in ChAc and MLS patients shows caudate nucleus and putamen atrophy, significant grey matter density reduction in the caudate head, and lateral ventricle dilation (Henkel et al. 2006; Huang et al. 2022; Jung, Hergersberg, et al. 2001; Peikert et al. 2002). Altered levels of sphingolipids and phospholipids in the caudate nucleus suggest that lipid processing deficits may be associated with the neuropathophysiology of ChAc (Miltenberger‐Miltenyi et al. 2023). ChAc patients exhibited bilateral symmetrical hypodensity of the pallidum, substantia nigra, and red nucleus on T2 and sense‐weighted MRI, indicating mineral deposition possibly due to enzyme defects from VPS13 gene mutations (Kaul et al. 2013). In MLS patients, the degree of basal ganglia atrophy and striatal hypometabolism correlates with the disease duration (Jung, Hergersberg, et al. 2001). We found that all ChAc patients, and half of MLS patients with cranial MRI suggested caudate and putamina atrophy, and although the age of onset and mean age of ChAc patients were lower than that of MLS, caudate atrophy was more common, which may help in the diagnosis of both MLS and ChAc, and is worth studying in the future with an expanded sample.

### 4.5 Limitations and Future Directions

There are several limitations to this study. First, this was a single‐center study based on local patients.

Second, due to limitations in technology, finances, and resources, we were unable to perform Western blot testing for VPS13A on the patients. Therefore, we could not directly assess the impact of these mutations on the expression and function of chorein in these patients, a limitation observed in other studies as well (Chen et al. 2023). Due to certain patient‐specific factors, we were unable to confirm McLeod phenotype for all patients in the absence of proper Kell antigen analysis. For the three cases with *XK* mutations plus extra‐hematologic features of MLS, it is sufficient to assume the presence of the Kell McLeod phenotype for practical management. The confirmation of McLeod phenotype is important both for proving the pathogenicity of the mutation and for being prepared for the potential risks associated with blood transfusions and the need for prophylactic blood banking. Routine sequencing of the *XK* and *VPS13A* genes should be considered in patients presented with choreatic movements even in the absence of acanthocyte studies (after HD was excluded), which would contribute to the early diagnosis of MLS and ChAc.

## 5 Conclusion

In conclusion, we reported eight Chinese MLS and ChAc families and identified two novel *XK* variants in three index MLS patients and six novel *VPS13A* variants in five index ChAc patients. One copy number variant of *VPS13A* was firstly described in Chinese population. Our study expands the genetic spectrum of *XK* and *VPS13A* and helps the clinical diagnosis of MLS and ChAc.

## Author Contributions

Data acquisition, analysis, and interpretation, and manuscript preparation were performed by Hao Yu, Ling Li, Xiaoyan Li, and Haipeng Liu. Hao Yu contributed to the study design and conception and critical revision of the manuscript. The first draft of the manuscript was written by Hao Yu, Ling Li, and Haipeng Liu and all authors commented on previous versions of the manuscript. All authors read and approved the final manuscript.

## Ethics Statement

This study was approved by the Ethics Committee of the Second Affiliated Hospital of Zhejiang University School of Medicine.

## Consent

Written informed consent was obtained from all patients participating in the study.

## Conflicts of Interest

The authors declare no conflicts of interest.

## Supporting information


Table S1.

## Data Availability

The data used and analyzed in the study are available from the corresponding author on proper request.
