# Supplementary material for: Clinical Features and Novel Pathogenic Variants of Chinese Patients With McLeod Syndrome and Chorea‐Acanthocytosis
Source: Mol Genet Genomic Med. 2024 Sep 26;12(9):e70015. doi: 10.1002/mgg3.70015 (PMC11425086; doi:10.1002/mgg3.70015)
Supplement: Supplementary file 1 — Table S1. [file MGG3-12-e70015-s001.docx]

**Table S1** List of genes responsible for related diseases of chorea and dystonia

| No. | Disease | Gene | OMIM |  | No. | Disease | Gene | OMIM |  | No. | Disease | Gene | OMIM |
| --- | --- | --- | --- | --- | --- | --- | --- | --- | --- | --- | --- | --- | --- |
| 1 | CHAC | *VPS13A* | 200150 |  | 19 | DYT1 | *TOR1A* | 128100 |  | 37 | WD | *ATP7B* | 277900 |
| 2 | MCLDS | *XK* | 300842 |  | 20 | DYT2 | *HPCA* | 224500 |  | 38 | FDFM | *ADCY5* | 606703 |
| 3 | HDL1 | *PRNP* | 603218 |  | 21 | DYT3 | *TAF1* | 314250 |  | 39 | LNS | *HPRT1* | 300322 |
| 4 | NBIA1 | *PANK2* | 234200 |  | 22 | DYT4 | *TUBB4A* | 128101 |  | 40 | Alpha-methylacetoacetic aciduria | *ACAT1* | 203750 |
| 5 | NBIA2B | *PLA2G6* | 610217 |  | 23 | DYT5a | *GCH1* | 128230 |  | 41 | GA I | *GCDH* | 231670 |
| 6 | NBIA3 | *FTL* | 606159 |  | 24 | DYT5b | *TH* | 605407 |  | 42 | Methylmalonic aciduria | *MUT* | 251000 |
| 7 | NBIA4 | *C19orf12* | 614298 |  | 25 | DYT6 | *THAP1* | 602629 |  | 43 | Propionicacidemia | *PCCA* | 606054 |
| 8 | NBIA5 | *COASY* | 615643 |  | 26 | DYT8 | *PNKD* | 118800 |  | 44 | Propionicacidemia | *PCCB* | 606054 |
| 9 | NBIA6 | *WDR45* | 300894 |  | 27 | DYT10 | *PRRT2* | 128200 |  | 45 | HPABH4A | *PTS* | 261640 |
| 10 | Aceruloplasminemia | *CP* | 604290 |  | 28 | DYT11 | *SGCE* | 159900 |  | 46 | HPABH4C | *QDPR* | 261630 |
| 11 | SPG35 | *FA2H* | 612319 |  | 29 | DYT12 | *ATP1A3* | 128235 |  | 47 | GM1-gangliosidosis | *GLB1* | 230650 |
| 12 | KRS | *ATP13A2* | 606693 |  | 30 | DYT16 | *PRKRA* | 612067 |  | 48 | HLD6 | *TUBB4A* | 612438 |
| 13 | WDSKS | *DCAF17* | 241080 |  | 31 | DYT18 | *SLC2A1* | 612126 |  | 49 | Aromatic L-amino acid decarboxylase deficiency | *DDC* | 608643 |
| 14 | IBGC1 | *SLC20A2* | 213600 |  | 32 | DYT23 | *CACNA1B* | 614860 |  | 50 | HMNDYT1 | *SLC30A10* | 613280 |
| 15 | IBGC4 | *PDGFRB* | 615007 |  | 33 | DYT24 | *ANO3* | 615034 |  | 51 | PKDYS1 | *SLC6A3* | 613135 |
| 16 | IBGC5 | *PDGFB* | 615483 |  | 34 | DYT25 | *GNAL* | 615073 |  | 52 | THMD2 | *SLC19A3* | 607483 |
| 17 | IBGC6 | *XPR1* | 616413 |  | 35 | DYT26 | *KCTD17* | 616398 |  | 53 | MTS | *TIMM8A* | 304700 |
| 18 | DRD | *SPR* | 612716 |  | 36 | DYT27 | *COL6A3* | 616411 |  | 54 | SPAX2 | *KIF1C* | 611302 |
